# Treatment of Pulmonary Phthisis—I

**Published:** 1893-07-15

**Authors:** 


### CITY OF LONDON HOSPITAL FOR DISEASES OF THE CHEST, VICTORIA PARK.—I.

### Treatment of Pulmonary Phthisis.

The first consideration in the management of consumptive patients is to insure good hygienic surroundings, not only as an important factor in arresting the disease itself, but also as a protection to others from possible infection. A brief description of the means employed at this hospital may therefore be of service, if only to indicate the points to be observed.

*Ventilation, Temperature, &c.*—The hot air supply is generally sufficient to maintain the desired temperature, but is reinforced in the corridor and larger wards by hot-water cock, which can be shut off if necessary, and by fireplaces, the use of which is seldom required. In the daytime it is endeavoured to keep the temperature as uniformly as possible at 60 to 61 deg. F., but at night, when all the patients are in bed, it is allowed to fall to 58 or 57 deg. The temperature in each ward is recorded by the nurses every four hours, attention being thus drawn at once to any deficiency in the heating apparatus.

*Prophylactic Measures, Disposal of Sputum, &c.*—Each patient is provided with a porringer, of about one pint capacity, containing four or five ounces of a solution of carbolic acid (1 in 40). Spittoons containing the same solution are placed in the wards, corridors, day-rooms, lavatories, and waiting-rooms in the out-patient department, and there are printed rules, strictly enforced, enjoining patients to expectorate in these and nowhere else. The spittoons are emptied and cleaned once a day, the porringer twice or oftener, the contents being poured down a sink used specially for this purpose. It is not contended that a carbolic acid solution of this strength exercises a completely destructive action on the tubercle bacilli, but it serves the purpose of a deodorant, and, what is much more important, keeps the sputum moist, experiments having shown that infection is only likely to arise from dried secretions. For this reason, pockethandkerchiefs should be frequently changed and disinfected, and not allowed to lie under the pillow or in the patient's locker. A small wire basket, somewhat similar to a sponge basket, has lately been found to answer the purpose here, and is hooked on to the bed-head within easy reach of the patient. A piece of lint or old linen, which can be burnt after a few' hours use, may be substituted for the handkerchief with advantage. Patients, especially children, are warned not to swallow their sputum, as by doing so infection may be carried directly to the intestines.

Acute and advanced cases are isolated as much as possible in the smaller wards, and this is a point of special importance in the case of children, who, being generally favourites with the older patients, may be rendered liable to infection by kissing. A child with non-tubercular disease is not placed in the same ward as a consumptive one if possible. The above precautions are of course based on the theory that consumption is an infective disease, but in any case their adoption is at least conducive to cleanliness.

*Direct Treatment.*—For the sake of convenience, this will be described as it is carried out in the acute or the chronic stages, although many cases occupy a doubtful position between the two. Symptomatic treatment will be dealt with under separate headings.

*Acute Phthisis.*—Cases of early phthisis, in whom pyrexia is a marked symptom, or chronic cases with exacerbations of temperature from time to time, may be discussed under this heading.

The patient must be kept absolutely at rest in bed in a well ventilated ward, care being taken to avoid draughts. The temperature of the ward should be kept as uniformly as possible from 59 deg. to 60 deg. F., and the amount of bedclothes regulated chiefly by the patient's sense of comfort.

The diet is a question of chief importance, and must be chosen with reference to the condition of the stomach; it should be light and easily assimilable, and given in small quantities at frequent intervals. Milk, diluted with soda-water or lime-water, and beef tea constitute the staple diet in this condition, but this may be modified according to circumstances. If beef tea cause diarrhœa, mutton or chicken broth may be substituted, or one of the numerous preparations of meat extract. Lightly boiled eggs or boiled fish may be given with caution if they can be taken without discomfort. Stimulants are not required in early stages unless there is much dyspnœa or prostration. If the disease has commenced with hæmoptysis, milk and ice should constitute the sole diet until all traces of blood in the sputum have disappeared. The further treatment of hæmoptysis is described separately.

With regard to drugs, the indications are mainly symptomatic, but, as will be mentioned presently, creasote sometimes seem to exercise a beneficial influence. The hypophosphites are apparently of service in some cases where the physical signs indicate a catarrhal condition of the lungs. Hypophosphite of soda gr. v. sod., bicarb. gr. v., spirit-chlorof. ℳx., tinct. cardamom ʒgr., and aq. camph. ad. ℥i., is a mixture frequently used here, and appears to exert a beneficial influence on the stomach. If there is a tendency to hæmoptysis, sweating, or diarrhœa, the calcium salt is used, prescribed as follows: ℞. calcii. hypophosph. gr. v., liq. calcii. sacch. ℳx., glycerini ℳxv., aq. camph. ad. ℥i., to be given three times a day. Hypophosphite of lime is sometimes given in larger doses of gr. xv. with advantage.

Antipyretics are occcasionally beneficial, but, are on the whole, of somewhat doubtful value in this disease, not only on account of the depression and gastric disturbances to which they are apt to give rise, but also for the reason that reduction of temperature so induced is by no means invariably followed by improvement in other respects. Moreover, pyrexia in phthisis is seldom accompanied by delirium or other cerebral symptoms, and, with the exception of sweating, it is often remarkable to how little discomfort a temperature of 103 deg. or even higher will give rise. It must be remembered in giving antipyretics that the abnormal temperature is a result and not the cause of the disease, and unless distinct and general improvement follows this line of treatment it should be at once discarded. It is usual here to try the effect of antipyretics when there is a more or less continuous temperature of 102·5 deg. or higher, or when it is of a hectic type, with a maximum of 103 deg., and followed by profuse sweating.

In the former case the sulphate of quinine, in doses of three to ten grains (varying with the age and toleration of the patient), may be given three or four times a day, together with acid sulph. dil. ℳx., syrup. aurant ʒi, aq. chlorof. ad. ℥i, and if the temperature is thereby lowered, it may be continued so long as there are no symptoms of cinchonism. In some cases larger doses, given once or twice a day, are more effective. When there is marked remission or intermission, the same drug in doses of five to ten grains may be given two hours before the time at which the maximum temperature usually occurs. The temperature should be recorded at least every four hours in all cases in which antipyretics are given, and the time of administration of the drug written down on the chart. Antipyrin (gr. xv.) is sometimes given instead, but its frequent use has a very depressing effect, and in phthisis especially is apt to give rise to its characteristic eruption. Antifebrin (gr. v.) may also be substituted for quinine, being equally efficacious, and in most cases less depressing than antipyrin. Phenacetine has been recently used here with apparent benefit, in doses of gr. x. to xx., given twice or three times a day. It is most effectual in lowering the temperature, and appears to exert few subsequent ill effects.

*Antiseptics.*—Inhalations of some volatile antiseptic solution are given as a matter of routine to all phthisical cases, whether acute or chronic. The respirator used is the one devised by Dr. Burney Yeo, consisting of a perforated metal cone, containing at its apex a small piece of sponge on which the fluid to be inhaled is poured. The solution most generally used here consists of equal parts of creasote and alcohol, about ten drops being poured on the sponge twice or three times a day. The oil of Pinus Sylvestris may be substituted for creasote; and another useful solution consists of olei eucalypti 10 c.c., chloroformi 2 c.c., spir. vini rect. 10 c.c., iodoformi 1 gr.

Patients are encouraged to wear the respirator, generally known by them as the "muzzle," for several hours a day, in fact, as often as they can conveniently do so, and many patients can wear it comfortably while sleeping. It is not contended that this form of treatment exerts any curative action on the disease, but it is certainly a valuable palliative remedy, rendering the cough less irritating, and the sputum and breath less offensive, and is therefore of service to others, even if not to the patient himself.

Antiseptics are also given internally, the one most frequently used here being creasote. It may be prescribed in pill, *e.g.*, creasoti ℳj, picis pan q. s., or in capsules, which are more tasteless, containing one or two minims, one of either to be given three or four times a day. The following mixture is also an excellent mode of administering it: ℞. creasoti (ℳ i. to v.), spiritus vini rect. (ℳx), aq. camph. ad. ℥i. This is given three times daily, beginning with one minum of creasoti and gradually increasing the dose until four or five minums are reached. Some cases of early acute phthisis have gained marked benefit under this drug, and it is being increasingly used in this condition. It appears to exert a gradually favourable influence on the pyrexia and sweating, and in most cases increases the appetite. The above mixture is easily taken, and rarely gives rise to nausea or other digestive disturbances, but the latter when present contra-indicates its use, and the capsules may be substituted. In more advanced cases where excavation has occurred, and where the sputum is profuse and offensive, creasote is also indicated, but here its good effects are necessarily not so obvious as in earlier stages.

In cases which proceed favourably, treatment becomes gradually that of the chronic stage. The diet is cautiously increased, fish or chicken being given first, and finally full meat diet. When the temperature is normal, or nearly so, the patient may be allowed to leave his bed for an hour or two, and if with no bad results for a gradually increasing period. The further treatment of such cases is chiefly a matter of hygiene, and they are, if possible, sent to a convalescent home for a few weeks, and warned against unhealthy occupations and habits of life, but in the case of unfitted patients, it is of course useless to give elaborate instructions which circumstances prohibit them from carrying out.

Acute outbreaks frequently occur in the course of chronic phthisis, probably due to a fresh deposit of tubercle in a hitherto unaffected part of a lung. The treatment is constructed on the lines given above, but speaking generally, the rules with regard to diet and rest in bed are not so rigorously enforced. In such cases the temperature may never become quite normal, and owing to the more advanced stage of the disease the chances of arrest are more remote. In the absence of hæmoptysis, diarrhœa, or other complication, the diet may be a generous one, with or without stimulants, and it is generally conducive to sleep if the patient is allowed up for an hour or two. Drugs are required chiefly with reference to the stomach, as described below, or creasote may be given. In some cases of advanced phthisis with cavitation, examination of the sputum shows the presence of numerous putrefactive organisms, and therefore it is possible that the pyrexia may be more the result of septicæmia than of a specific tubercular inflammation. In such cases creasote or quinine may be of service, and stimulants are often beneficial. More rarely in the course of chronic phthisis exaltation of temperature is the result of a simple brocho-pneumonia of two or three weeks duration, not due to tubercular infiltration, as shown by subsequent disappearance of the physical signs. Absolute rest, easily assimilable food, and poultices to relieve pain are the chief points in the treatment, and hypophosphite of lime, as prescribed above, is especially used in this condition.

